# Proteomics of *Cryptococcus neoformans*: From the Lab to the Clinic

**DOI:** 10.3390/ijms222212390

**Published:** 2021-11-17

**Authors:** Ben Muselius, Shay-Lynn Durand, Jennifer Geddes-McAlister

**Affiliations:** Molecular and Cellular Biology Department, University of Guelph, Guelph, ON N1G 2W1, Canada; bmuseliu@uoguelph.ca (B.M.); sduran01@uoguelph.ca (S.-L.D.)

**Keywords:** *Cryptococcus neoformans*, mass spectrometry-based proteomics, fungal pathogenesis, in vitro and in vivo models, personalized medicine

## Abstract

Fungal pathogens cause an array of diseases by targeting both immunocompromised and immunocompetent hosts. Fungi overcome our current arsenal of antifungals through the emergence and evolution of resistance. In particular, the human fungal pathogen, *Cryptococcus neoformans* is found ubiquitously within the environment and causes severe disease in immunocompromised individuals around the globe with limited treatment options available. To uncover fundamental knowledge about this fungal pathogen, as well as investigate new detection and treatment strategies, mass spectrometry-based proteomics provides a plethora of tools and applications, as well as bioinformatics platforms. In this review, we highlight proteomics approaches within the laboratory to investigate changes in the cellular proteome, secretome, and extracellular vesicles. We also explore regulation by post-translational modifications and the impact of protein–protein interactions. Further, we present the development and comprehensive assessment of murine models of cryptococcal infection, which provide valuable tools to define the dynamic relationship between the host and pathogen during disease. Finally, we explore recent quantitative proteomics studies that begin to extrapolate the findings from the bench to the clinic for improved methods of fungal detection and monitoring. Such studies support a framework for personalized medical approaches to eradicate diseases caused by *C. neoformans*.

## 1. Introduction

Combatting infectious diseases and overcoming drug-resistant pathogens is critical for global health and food security. In humans, fungal pathogens represent substantial threats to global health, with more than 300 million people afflicted by serious fungal diseases annually [1]. Such infections cause over 1.6 million deaths with many deaths occurring in resource-limited regions, where support is needed the most [1,2]. In recent years, the frequency of invasive fungal infections has increased by over 200% with patient mortality rates ranging from 30 to 90%, depending on the pathogen, diagnosis, treatment regimes, and patient immune status [3]. Among immunocompromised individuals, fungal infections are particularly prevalent, including individuals suffering from HIV/AIDS, cancer patients receiving immunotherapy, organ transplant recipients administered immunosuppressive drugs, as well as the ever-increasing elderly population [2,4]. The treatment of fungal infections is a serious challenge given our limited selection of low-toxicity, clinically effective antifungal drugs, as well as the evolution and emergence of antifungal-resistant strains [5,6]. Of particular interest is the widespread human fungal pathogen, *Cryptococcus* spp., which has demonstrated resistance against all four classes of antifungals (i.e., azoles, polyenes, allylamines, and echinocandins), global distribution, and complex pathogenesis towards both immunocompromised and immunocompetent individuals, underscoring the immense need for innovative treatment strategies [6,7].

To identify proteins and pathways that are relevant to fungal pathogenesis and modulation of the host immune response, and to discover new treatment strategies, mass spectrometry-based proteomics provides a sensitive and robust platform [8]. Specifically, differences in protein abundance from both the host and pathogen perspectives can be explored in a single experiment. Additionally, assessment of global cellular remodelling under different growth conditions, during infection, and between pathogenic species and strains can be quantified. Broadly speaking, mass spectrometry-based proteomics is divided into top–down vs. bottom–up proteomics, with the former assessing intact protein complexes and proteoforms, and the latter sequencing detected peptides of digested proteins to provide information on cellular regulation [9,10]. For quantification of proteins, several methods using relative or absolute approaches are widely available, including label-free quantification (e.g., peak intensity and spectral counting), metabolic labelling (e.g., stable isotope labelling with amino acids in cell culture [SILAC]), or chemical labelling (e.g., tandem mass tags [TMT] and isobaric tag for relative and absolute quantification [iTRAQ]) [11]. As demonstrated in this review, the applications of proteomics technologies are broad and the methods for sample preparation, quantification, and bioinformatic processing are diverse and dynamic, lending themselves to tailored and powerful profiling of biological systems. Moreover, the application of mass spectrometry-based proteomics within the clinic enables the analysis of solid tissues, cells, and liquid samples for detection of protein presence and abundance within the samples of interest. This information supports the development of precision medicine to diagnosis disease states and predict treatment trajectory or drug responsiveness [12]. Exploring a timeline of mass spectrometry in clinical laboratories defines early beginnings with applications in drug screens, metabolism and hormones measurements, and microbial identifications [13]. More recently, proteomics investigates preclinical cohorts, and profiles plasma and biomarkers in a variety of diseases, as well as leveraging public date for precision medicine programs [14,15,16,17,18].

Bottom–up mass spectrometry-based proteomics of disease, the focus of this review, provides insight into disease mechanisms and the host’s ability to fight against infection, along with the discovery and characterization of novel drug targets [19,20]. For example, the role of transition metal acquisition (e.g., zinc) on the intracellular and extracellular environments of *Cryptococcus neoformans* was recently explored using quantitative proteomics [21]. In the study, information about regulated modulation of the pathogen during replete vs. limited conditions was revealed and the authors investigated an uncharacterized protein (i.e., Wos2) with roles in virulence factor production. This approach provides new insight into fungal pathogenesis, as well as opportunities to identify proteins with previously undefined roles as potential new targets for antifungal design. Proteomics analyses can also be combined with other OMICs technologies (e.g., genomics, transcriptomics, metabolomics, and lipidomics) to profile a multitude of molecular levels and gain further insight into regulatory patterns [22]. Recently, a combinatorial method for multimolecular OMICs profiling for systems biology was developed (i.e., simultaneous metabolite, protein, lipid extraction [SIMPLEX]) providing a simple and effective method for consistent and robust analyses [23]. Importantly, the role of bioinformatics tools in compiling, processing, analysing, integrating, and visualising such complex data is critical to the application of proteomics methodology and supports the enhancement of currently available platforms. To date, application of SIMPLEX for infectious disease research, and in particular for fungal studies, has been limited, but it demonstrates an exciting advancement in OMICs abilities and warrants further investigation for new insights into disease and host immunity. 

**Figure 1:**
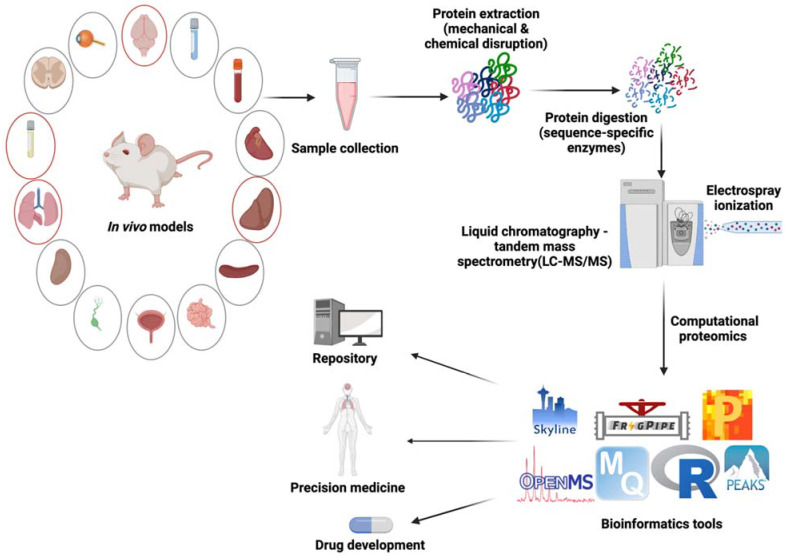
Mass spectrometry-based proteomics for in vivo profiling of *C. neoformans* infection. Focusing on murine models of infection, several tissues and fluids have been profiled by LC-MS/MS (circled in red), including the brain, lungs, liver, and cerebral spinal fluid. Additional tissues and fluids for exploration (circled in grey), given the dissemination of fungal cells throughout the host, include: the spinal cord, eye, spleen, small intestine, heart, bladder, lymph nodes, kidney, blood, and lung lavage. Upon sample collection, proteins are extracted with mechanical (e.g., grinding) and chemical (e.g., detergent) strategies followed by digestion of proteins into peptides with sequence-specific enzymes (e.g., trypsin and Lys-C). Peptides are then ionized (e.g., electrospray ionization) and measured on a mass spectrometer (e.g., liquid chromatography-tandem mass spectrometry) followed by data analysis using a suite of bioinformatics platforms (e.g., MaxQuant, Perseus, FragPipe and MSfragger, OpenMS, Skyline, PEAKS, and R) [24,25,26,27,28,29]. The acquired data can inform a variety of downstream applications, including drug development, precisions medicine, and construction of repositories for data sharing. Figure generated with Biorender.com (accessed 12 November 2021).

In this review, we present the role of mass spectrometry-based proteomics in defining and exploring the widespread human fungal pathogen, *C. neoformans*. We begin by providing background about the fungal pathogen, disease determinants, treatment options, and the rise of antifungal resistance. Next, we emphasize in vitro studies that provided a foundation for our current knowledgebase through proteomic profiling of the cellular proteome, secretome, vesicles, regulatory networks, and interactions between the pathogen and host cells. Moving beyond the in vitro data, we explore the plethora of in vivo models developed and assessed for cryptococcal infection and highlight the vast opportunities to leverage these models for an in-depth analysis of the disease. This approach allows us to move beyond our laboratory knowledge and explore the potential of in vivo datasets within the clinic with an eye towards personalized medicine to diagnose, treat, and track fungal infections (Figure 1). The purpose of our review is to feature a medically relevant fungal pathogen, and suggest potential proteomic clinical applications, along with avenues to uncover novel therapeutic strategies, to combat infection on a global scale.

## 2. Defining the Fungal Pathogen—*Cryptococcus* spp.

### 2.1. Distribution and Disease

The primary causal agent of cryptococcosis within immunocompromised individuals is *C. neoformans*, accounting for more than 220,000 infections and 181,000 deaths worldwide each year, and within immunocompetent individuals, *Cryptococcus gattii* [30]. The latter being associated with an outbreak along the northwest coast of the United States and Canada since 2009 [31]. The fungi are found ubiquitously within the environment (e.g., decaying vegetation and bird faeces), causing infection through inhalation of desiccated fungal cells, and evading the host immune system through colonization [7,32]. Invasion is followed by proliferation within alveolar macrophages in the lung, resulting in pulmonary cryptococcosis. Subsequently, fungi can disseminate throughout the body, cross the blood–brain barrier, and invade the central nervous system, resulting in fatal cases of cryptococcal meningitis and meningoencephalitis [33,34]. Within immunocompromised individuals, a reduced immune function worsens disease prognosis by increasing the chance of spread to the central nervous system. Importantly, dissemination of *C. neoformans* to the brain vasculature is highly correlated with HIV/AIDS mortality rates, and despite current treatment options, approximately 20% of all cryptococcosis cases remain fatal [35]. These tragic outcomes are often correlated with low regional income, poor diagnostics, and limited antifungal availability.

### 2.2. Virulence Factors

To promote fungal survival and proliferation within the human host, *Cryptococcus* spp. rapidly adapts through production of specific virulence factors [4,24,25,26]. These determinants serve dual proposes to prevent microbial recognition by the host immune system and to inflict host damage. For example, elaboration of a polysaccharide capsule protects the fungus from the harsh environmental conditions of its host, prevents phagocytosis by innate immune cells, and promotes intracellular proliferation [27]. The fungus also produces melanin to protect fungal cells from UV radiation, provide structural integrity, and promote survival at extreme temperatures, along with mechanisms regulating thermotolerance for survival at mammalian body temperature (i.e., 37 °C) [28]. Moreover, the fungus releases and actively secretes extracellular enzymes, such as proteases and urease, which damage brain endothelial cells to increase permeability of the blood brain barrier and promote invasion of the central nervous system [29]. Lastly, the fungus secretes vesicles to enhance pathogenesis by transporting capsule precursors and secreted enzymes throughout the host [30]. The regulation of these virulence factors is dominated by the well-characterized cAMP/Protein kinase A (PKA) signalling pathway [31,32]. Importantly, many questions remain about the mechanisms used by the fungus to evade the host immune response, as well as strategies used by the host for protection and clearance of invading pathogens.

### 2.3. Host Immune Response

The initial line of defence against an invading fungal pathogen is both physical and cellular. For instance, upon inhalation of fungal desiccated cells, the lung epithelial cells provide a physical barrier and initiate a programmed defence response [36,37,38]. This response activates the innate immune systems to induce production of lung surfactant proteins that attract eosinophils, initiate phagocytosis by alveolar macrophages, and prime the complement cascade. Notably, within alveolar macrophages, fungal vesicles containing capsule polysaccharide can accumulate, impairing host cellular function (e.g., protein synthesis and cell cycle) and disrupting membrane repair mechanisms [39]. The primary adaptive immune response to *Cryptococcus* spp. is stimulation of CD4+ T lymphocytes by dendritic cells, which secrete cytokines to recruit other lymphocytes and phagocytes to the site of infection [40]. When immunocompromised individuals have reduced immunity (e.g., impairment of CD4+ T cells), fungal cells can disseminate throughout the body, directly damaging effector cells of the immune system and impacting the host’s ability to execute an effective response [41]. Importantly, in immunocompetent individuals, formation of granulomas (i.e., structures composed of several cell types, such as dendritic cells, macrophages, T cells, and fibroblasts) in the lungs, work together to contain the pathogen and prevent dissemination [42]. A deeper understanding of the complexities between fungal survival and immune system regulation is needed to unveil opportunities to enhance the immune response and clear infection.

### 2.4. Antifungal Treatment and Resistance

Strategies to treat cryptococcosis depend on multiple factors, including host immunodeficiencies, fungal strain properties, antifungal availability, and disease localization [37]. Notably, the design and development of antifungal drugs is challenging given the close evolutionary relationship between humans and fungi, which translates to an overlap of targets and common cytotoxicity profiles between the biological systems [5,6,7]. Effective antifungals demonstrate selective toxicity towards fungal cells (e.g., fluconazole), which penetrate the cerebral spinal fluid and displays minimal host toxicity. However, the requirement for prolonged administration (e.g., 6–12 months) supports the development of resistance. Another widely used antifungal, Amphotericin B, treats infections of the central nervous system, but challenges with host toxicity requires intravenous administration, limiting its application in developing nations. Combination therapy (e.g., Amphotericin B and Flucotysine) is an alternative approach and is often recommended to limit the development of resistance. However, limitations of short half-lives and the requirement for excess concentrations administered over repetitive treatments, also increases the risk of resistance. The need for new treatment options and an understanding of mechanisms driving antifungal resistance is great and mass spectrometry-based proteomics provides a platform for such investigations.

## 3. Proteomic Profiling of Cryptococcal Infection In Vitro

**Figure 2:**
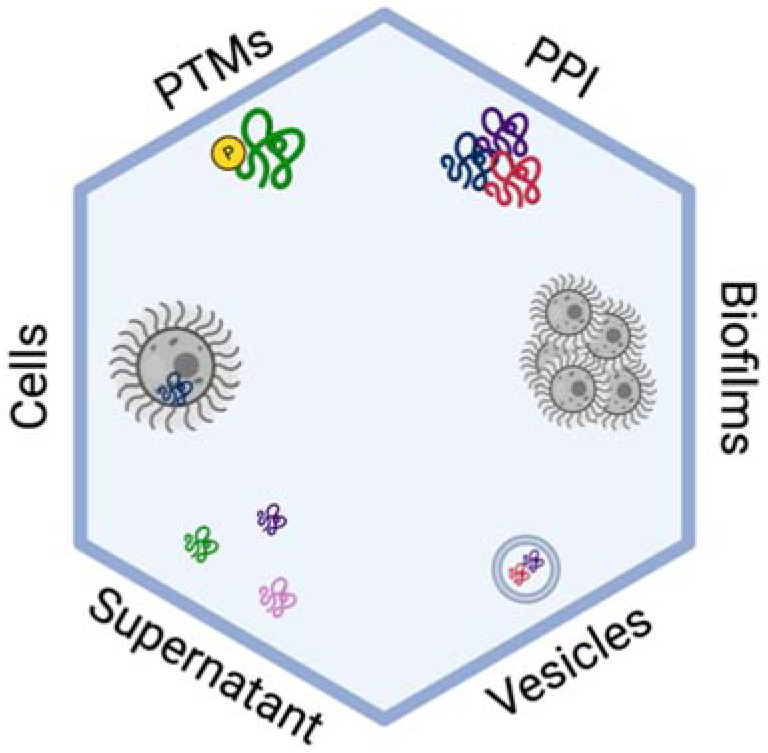
Mass spectrometry-based proteomics for in vitro profiling of *C. neoformans*. Highlighted studies define changes in the cells (i.e., cellular proteome), supernatants (i.e., secretome), vesicles (i.e., extracellular), biofilms (i.e., collection of fungal cells), PTMs—post-translational modifications (e.g., phosphorylation), and PPI—protein–protein interactions (e.g., complex formation). Figure generated with Biorender.com (accessed 12 November 2021).

Applications of mass spectrometry-based proteomics to define virulence mechanisms and profile cellular remodelling of *Cryptococcus* spp. have made substantial contributions over the past 15 years. In this section, we present foundational in vitro studies and their potential for extrapolation within in vivo models (Figure 2). For example, proteomic investigation of cAMP/PKA pathway regulation in *C. neoformans* uncovered a novel drug repurposing strategy [43]. In this study, significant differences in production of 302 proteins were identified between the wild-type *C. neoformans* serotype A (H99) strain and a *pka1*Δ using dimethyl labelling (i.e., stable isotope labelling of peptides) for quantification combined with single-run mass spectrometry on a linear-trapping quadrupole orbitrap instrument (Linear trap quadrupole [LTQ]-Orbitrap Velos) [44]. Processing and analysis of the mass spectrometry data was performed using MaxQuant with the Andromeda plug-in and Perseus platforms, complemented with network mapping via STRING (Search Tool for the Retrieval in Interacting Genes/Proteins) [24,25,45,46]. This proteome profiling identified a new role for the ubiquitin proteasome pathway in capsule regulation and reported the use of bortezomib against the fungal pathogen. Additionally, secretome profiling following PKA regulation in *C. neoformans* identified secreted virulence factors influenced by the absence of Pka1 and discovered the first biomarkers of cryptococcal infection using an in vivo model [47]. Here, a time course of protein secretion was observed between WT and *pka1*Δ strains, following reductive demethylation for protein quantification, followed by protein measurement on an orbitrap mass spectrometer (LTQ-Orbitrap Velos). Proteomic profiling identified five proteins with significant changes in abundance, which were then traced through macrophages and murine blood samples following cryptococcal infection using multiple reaction monitoring (MRM) in the presence of stable isotope dilutions for identification and quantification of the selected peptides. Together, these studies explored dynamic signalling mechanisms and uncovered opportunities for moving the discoveries into the clinic with a novel diagnostic tool and treatment option.

An array of proteomics studies also advanced our knowledge of key virulence regulators, such as extracellular vesicles, and structures of clinical relevance (i.e., biofilms). For instance, a review of early proteomic profiling of extracellular vesicles from a variety of fungal pathogens (e.g., *C. neoformans*, *Histoplasma capsulatum*, and *Saccharomyces cerevisiae*) described the role of proteomics in distinguishing conventionally vs. unconventionally secreted proteins within the vesicles, including proteins with both immunological and pathogenic activities [48]. Additionally, information pertaining to protein function and biogenesis, as well as the expansion of proteomics data to highlight interconnectivity among fungal pathogens was covered. Primary research of extracellular vesicles investigated the morphology and roles of vesicles within *C. neoformans* using a combination of electron microscopy, proteomics, enzymatic activity, and serological reactivity [49]. Here, early adaptation of mass spectrometry-based proteomics to explore fungal pathogens relied on an orbitrap mass spectrometer (LTQ-Orbitrap XL) with a qualitative assessment of proteins. The results defined roles for fungal vesicles in virulence and protection against oxidative stress, which were supported by biochemical assays for laccase and urease, followed by immune recognition of proteins within vesicles by sera. These data provide a framework for future studies exploring the roles of proteins within fungal vesicles for pathogenesis.

Another example of mass spectrometry-based profiling in *C. neoformans* research was carried out, examining the effect of extracellular vesicles produced by bone marrow-derived macrophages (BMDMs) in response to *C. neoformans* infection. BMDMs were infected with *C. neoformans* and secreted vesicles were isolated for multi-OMICs analysis, including proteomics, metabolomics, and lipidomics [50]. Notably, a simultaneous metabolite, protein, and lipid extraction (MPLEx) was performed using chloroform–methanol to separate the phases and measure each sample individually [51]. For proteomic profiling, a Q-Exactive orbitrap mass spectrometer was used with datasets analysed by MaxQuant with label-free quantification parameters. The datasets were complemented with transcriptome profiling to comprehensively identify all components of the isolated vesicles. Relative to proteomics, a core protein set was found in common across all isolated vesicles while a secondary protein set differed depending on the conditions of infection (e.g., in vivo vs. in vitro). These findings demonstrate a proinflammatory shift and increased activation of immune related pathways in naïve macrophages exposed to vesicles. Conversely, in vivo testing showed mice treated with vesicles isolated from BMDMs prior to *C. neoformans* infection had a lower pulmonary fungal load but shorter survival time due to a heightened inflammatory response. This suggests vesicles as a key signalling mechanism during infection, identified with activated and naïve macrophages in vitro with potential wider reaching significance in vivo.

For fungal biofilms, analysis of proteome differences between biofilm and planktonic *C. neoformans* cells detected changes in metabolism, protein turnover, and global stress responses [52]. Specifically, biofilm-associated cells showed increased abundance of proteins related to oxidation–reduction, proteolysis, and response to stress, along with a reduction in abundance of proteins involved in metabolic processes, transport, and translation. Here, MudPIT (multidimensional protein identification technology) was used to eliminate gel separation and directly measure peptides within the mass spectrometer, such as an Orbitrap (LTQ-Orbitrap XL) instrument combined with analysis of mass spectra using SEQUEST [53,54]. This study enables comparison between fungal and bacterial biofilm proteomes for appreciation of conserved cellular remodelling patterns detected despite the initiating pathogen.

Defining changes in protein production influenced by post-translational modifications explores signalling cascades and identifies both direct and indirect downstream targets, furthering our understanding of protein regulation. For instance, comprehensive profiling of phosphorylation events in *C. neoformans* provided a molecular level view of virulence regulation and pathogenesis, including definition of kinase pathways critical for fungal proliferation in vitro [55]. This study was seminal in exploring the role of phosphorylation on *C. neoformans*, using high resolution mass spectrometry (LTQ-Orbitrap Velos) of TiO_2_-enriched phosphopeptides (i.e., selective enrichment and purification of phosphopeptides) [56,57]. Data searching and analysis was performed using MASCOT (Matrix Science), SEQUEST, and Proteome Discoverer (Thermo Scientific, Waltham, MA, USA). Notably, two enrichment strategies were used to capture phosphopeptides, including single pot enrichment from cell lysate vs. fractionation of protein lysate into six fractions by basic pH reversed-phase liquid chromatography (bRPLC). The analysis revealed 1540 phosphorylation sites in 648 proteins, including 45 kinases, with characterization of the proteins by Gene Ontology analysis. Further, assessment of specific phosphorylation motifs revealed enrichment of kinases within the cyclin dependent kinase, mitogen activated protein kinase, and protein kinase C and PKA pathways, supporting connections among phosphorylation, cellular regulation, and thermotolerance. Notably, the data was assessed in a qualitative manner, leaving room for future studies to add a layer of complexity by exploring quantitative changes in protein abundance profiles under regulation of specific phosphorylation events. 

From the host perspective, phosphoproteome profiling of murine macrophages following co-culture with *C. neoformans* H99 demonstrated differential regulation of proteins in the AMPK-autophagy initiation complex (AIC) network [58]. The AIC plays an important role in initiating autophagy in mammalian cells. The signalling pathways that regulate autophagy rely on kinase AMPK, which is activated by phosphorylation, increasing kinase activity of the AIC [59,60]. Semi-quantitative profiling was performed using label-free nano-flow LC/MS-MS for total proteome measurement, combined with Fe-NTA phosphopeptides enrichment (immobilized metal affinity chromatography (IMAC) for selective enrichment of phosphopeptides) [61,62] for the phosphoproteome analysis. Phosphoproteomic profiling, combined with Western blot and phosphospecific flow cytometry, identified increasing rates of phosphorylation in alveolar macrophages. Further, when host cells were depleted of AIC components, AMPKa phosphorylation was reduced within macrophages, demonstrating defects in initiation of autophagy. These findings convey the importance of protein activities on phosphorylation of AIC components and overall fungal clearance.

## 4. In Vivo Modelling of Cryptococcosis

The use of animal models is key to gaining a better understanding of *C. neoformans* pathogenesis and host immune response. In vivo infection assays provide an opportunity to study infection in a more complex environment under conditions that cannot be simulated in vitro, providing a representation of the disease in humans, and promoting translation of findings from the lab to the clinic. In this section, we present murine models of cryptococcal infection and their diverse applications. 

A fundamental study on in vivo models used clinical isolates of *C. neoformans* from the cerebral spinal fluid of HIV-infected individuals to assess strain virulence (i.e., low, intermediate, and high) based on mortality rates within a murine model [63]. Mice were inoculated intranasally and their progression to morbidity was monitored, with mortality rates compared between mouse models and the human patients. The data conferred low virulence in mice with higher survival rates in humans, supporting the recapitulation of human infection in a murine model. Another critical study considered the impact of mixed populations (i.e., multiple fungal strains) within a single host during infection [64]. Such instances may arise when a host acquires multiple strains of *C. neoformans* from the environment, or through in vivo evolution leading to endogenous replication of yeast cells. Here, flow cytometry to analyse ploidy and amplification of loci by PCR to determine culture genotypes identified 20% of patients with mixed infections. The results showed differing ploidy, serotypes, and mating types within the populations, along with differing localization and temporal presence within the host. These results suggest co-infecting strains may interact and influence the outcome of infection. Importantly, evaluation of such models by proteomics would permit profiling of the interaction between pathogens, such as HIV and *C. neoformans* or between fungal isolates.

Murine models also provide an opportunity to investigate the host response to infection with relation to sex and age. For instance, intravenous inoculation with *C. neoformans* of both male and female murine models over a time course of infection (i.e., six, eight, or ten days post inoculation) showed that by day six, cytokine levels in plasma and spleen were significantly higher in females than in males [65]. These data support altered immune defences and outcomes to disease influenced by the host. For proteomics investigations, sex differences remain to be explored but may provide crucial insight into sex-specific immune responses and the need for tailored treatment. Other factors influencing murine models and the potential for clinical proteomics investigations involve the mode of inoculum delivery. Given the initiation of human infection within the lungs, in vivo studies typically involve intratracheal or intranasal inoculations to recapitulate natural uptake from the environment. Another approach includes intravenous infection, which bypasses immune responses in the lung, but allows investigation of direct dissemination of fungal cells to the brain. A recent study compared these three modes of inoculation using immunofluorescence to assess the presence of antigen within brain tissue [66]. After seven days, intravenous infection models showed a greater number of yeast cells within the brain with no significant difference in brain fungal burden between models infected intranasally or intratracheally. However, at three days post infection, these models demonstrated the presence of a high yeast cell count in the brain, which accumulated just hours after initial infection. These data support further investigation of the molecular underpinnings regulating dissemination, fungal pathogenesis, and host response.

Consideration for the role of diverse innate immune cells is also important when assessing fungal virulence and host immune response. Applying proteomics techniques to investigate cell-specific responses may inform on strategies to prime immune cells for an efficient and effective response. For example, alveolar macrophages are the first line of defence against cryptococcal cell, but dendritic cells also play a critical role in cross-talk between the innate and adaptive immune systems. Recently, the function of dendritic cells in cryptococcosis was studied in vivo using fluorescently labelled live *C. neoformans* or heat-killed *C. neoformans* [67]. Single-cell suspensions of lung cells at three times points of infection were analysed by fluorescence microscopy and flow cytometry to examine the uptake of fungal cells by murine cells. The analysis showed that pulmonary dendritic cells phagocytosed both live and heat-killed fungi after one hour of incubation, which was confirmed by confocal microscopy of internalisation. Next, expression of a dendritic cell maturation marker (i.e., CD11c) was monitored to determine if dendritic cell expansion or infiltration occurred after infection. The findings demonstrated the importance of pulmonary dendritic cells for phagocytosis of *C. neoformans* in vivo and supported a model for assessment of unique immune cells’ roles in regulating infection. 

## 5. Assessing Proteome Changes of Fungal Infection In Vivo

**Figure 3:**
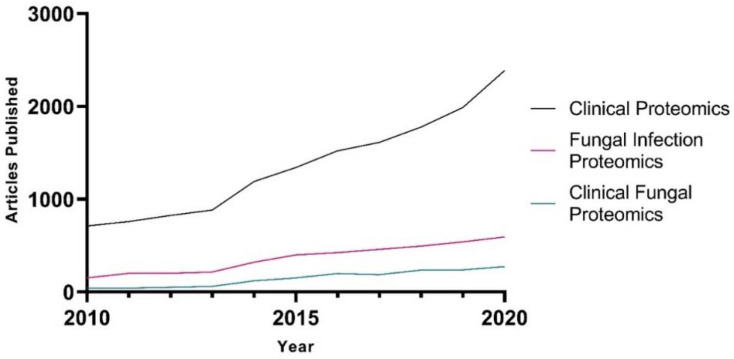
Publications from 2010 to 2020 of proteomics studying fungal infections and applications within the clinic. An in-house, developed R-script was used to search PubMed for publications using the following search terms within the abstract or title, or both: (i) clinical AND proteomics, (ii) proteomics AND (fungi OR fungal) AND infection, (iii) clinical AND (fungi OR fungal) AND proteomics.

With the establishment and assessment of diverse murine models of cryptococcosis, mass spectrometry provides an ideal tool to explore the intricate relationship between host and pathogen during infection using such models. In this section, we discuss the use of in vivo models of cryptococcal infection to investigate infectious disease; however, although various fungal strains and murine models are available, applications of proteomics within such models are limited, providing a valuable opportunity for further investigation. For instance, investigations often focus on the response of the host, given the excess abundance of host proteins relative to the invading pathogen, and is limited in detection of fungal remodelling. Focusing on the host substantially aids in our understanding of immunity but leaves many questions unanswered pertaining to the pathogen and its adaptability for survival within the host. Importantly, over the past two decades, the applications of mass spectrometry-based proteomics to investigate fungal pathogens, along with the use of proteomics in the clinic and for assessment of fungal infections within the clinic, are increasing, underscoring the value of improved accessibility of such strategies to combat fungal diseases (Figure 3).

Protein lysine acetylation (Kac) is a post-translational modification linking both histone and nonhistone acetylation and playing an important role in many pulmonary and neurological diseases, including cryptococcosis. A recent study investigated the mechanism of Kac modulation of host proteins following *C. neoformans* infection and explored how tissues differentially regulate Kac levels in response to infection [68]. Intranasal inoculation of six- to eight-week-old female mice with *C. neoformans* H99 was performed and, following seven days post infection, lung and brain tissues were collected for fungal load counts (i.e., infiltration of the lungs and dissemination to the brain), along with proteome and acetylome analysis. For quantification, 6-plex TMT labelling was performed, and for acetylome analysis, samples were incubated with anti-Kac pan antibody-conjugate beads to isolate peptides containing a Kac site. LC-MS/MS was performed on a hybrid quadrupole-orbitrap mass spectrometer (QExactive Plus) with the total proteome analysis used as a background control for quantifying acetylation alterations. This study showed that the acetylation status of histones and the transcription of chromatin are modulated by *C. neoformans* infection, which are important processes for the regulation of sugar metabolism, as well as host immune system expressions. Both the lung and brain tissue were found to have a similar number of acetylation sites; however, there was a lack of significant correlation between Kac detected in the lung and brain tissues. In the brain, Kac proteins were primarily lower in abundance; whereas, in the lungs, Kac proteins were higher in abundance. These data suggest that the brain and the lungs utilize different mechanisms to regulate acetylation and deacetylation. For further analysis, a multi-OMICs approach was taken, integrating of mRNA transcriptome data to compare gene regulation to protein production, supporting a significant correlation (R = 0.8042) between protein and mRNA level regulation. It is important to note that, while a significant correlation was observed, multiple cases of inverse regulation, as well as regulation only occurring at the protein or mRNA level, were also observed. This phenomenon has been well documented and investigated by prominent researchers in the field [69,70].

Comparative proteomics analysis between infected and uninfected, or among different treatment and growth conditions, can help tease apart specific responses of the host to fungal infection relative to the evaluated parameter. Such is the case for recent comparative profiling of *C. gattii* infection in the lungs of rats using mass spectrometry-based proteomics [71]. A bottom–up approach was used to analyse lungs from experimentally infected rats to identify host proteins with differential changes in abundance with findings validated in cell culture and in vivo models. Host changes associated with energy metabolism were observed, supporting the activation of glycolysis and lactate accumulation in lung cells. These results mimic a cancer-like metabolic status, defined as the Warburg effect. This effect involves an increase in the rate of glucose uptake and the preferential production of lactate, despite the presence and availability of oxygen [72]. Although this study focused on the host response, new insight into defence mechanisms regulated by *C. gattii* and the ability to define signatures of cryptococcal infection were uncovered.

Lastly, to detect proteins involved in cryptococcal invasion and infection within the human brain, proteomic profiling is a valuable strategy to study the interaction between host and pathogen to better understand disease progression and immune influences. For example, mass spectrometry-based proteomic analysis using iTRAQ combined with SCX fractionation measured changes in frontal lobe brain tissues from patients suffering from cryptococcal meningitis following co-infection with HIV [73]. The study identified over 300 differentially produced human proteins (close to 10% of the measured proteome) involved in the disease. As expected, elevation of immune-associated proteins (e.g., complement and major histocompatibility proteins), signal transduction cascades (e.g., caveolin), and extracellular matrix proteins (e.g., actin and collagens), involved in preventing fungal cell adhesion or aggregation, responded to infection. Overall, this study provides new mechanistic insight into co-regulation of immune responses and cryptococcal infection during HIV. Importantly, the study scope could be expanded to investigate the same questions in consideration of fungal proteins to define novel mechanisms of pathogenesis and potentially, uncover weaknesses in the fungal arsenal for exploitation. 

The next steps to support a framework for personalized medicine will move beyond reporting the in vivo model findings and towards the detection of specific proteins associated with disease. For example, the detection of host proteins involved in signalling and prevention of fungal aggregation could be stimulated by small molecules for enhanced activity during infection. Alternatively, the information gleaned from the host response to infection could propose novel anti-virulence strategies for drug design and development to limit the virulent activity of the pathogen without increasing selective pressure towards resistance. Lastly, multi-organ profiling of cryptococcal infection provides a roadmap of both host response and fungal progression, outlining potential biomarkers for measurement within the clinic to monitor the progression of disease and efficacy of treatments. Currently, proteomics in the clinic is heavily influenced by sample collection and preparation methods, reliability in sample storage and processing, instrument availability and sensitivity, consistency in operators, and data analysis. In addition, sharing the data with other researchers and clinicians and ensuring data availability and transparency is critical, as well as a consideration of ethics [74,75]. To date, many of the clinical proteomics guidelines are applicable within cancer research and neurological diseases but the opportunity to extrapolate such foundational work to infectious diseases is energizing and promising. 

## 6. Future Directions and Conclusions

In this review, we introduce the human fungal pathogen, *C. neoformans*, and highlight important in vitro studies, which provided the foundation for our knowledge about regulation of the cellular proteome, secretome, and vesicles, as well as cellular remodelling during biofilm formation. Additionally, we explore in vitro profiling of the host protein profile following co-culture with fungal cells and define the impact of post-translational modifications on both the fungi and host networks. We move beyond the bench and present intricate in vivo murine models of cryptococcal infection, which assess the influence of fungal strain on host survival, inoculation methods on fungal dissemination, the role of host sex and age on the immune response, and the importance of mixed pathogenic populations. Such in vivo models provide a framework for enhanced exploration of cryptococcal infections by advancing proteomics profiling beyond the lab and into the host. A few recent mass spectrometry-based proteomics studies profile changes in protein signatures following in vivo infection, providing new insights into host immunity and patterns of co-infection; however, it is limited in defining fungal responses. 

Here, we propose the value of extrapolating these studies to investigate cryptococcal infection from a dual perspective (i.e., both host and pathogen) in a plethora of tissues and fluids. The goal is to uncover infection parameters driven by temporal and spatial considerations using quantitative proteomics. A key limitation in clinical cryptococcal research are the currently available diagnostic tools, which often underdiagnose cryptococcosis due to similarities with other pulmonary diseases [37]. Recently, advances in lateral flow antigen detection testing for cryptococcal antigen permits pre-symptomatic testing and provides promise for advances in the clinic; however, precise monitoring of fungal burden, dissemination, and response to treatment is limited [76]. Therefore, by advancing the use of proteomics within the clinic (e.g., species identification, biomarker detection, diagnosis, and prognosis) we aim to map a signature of cryptococcal infection throughout the host. Such an approach encourages the detection and characterization of novel virulence factors, monitoring of treatment efficacy and changes in fungal burden, along with tailored host immune responses, which may be leveraged as new therapeutic options. Taken together, the strategies outlined in this review, combined with an eye towards the future, uncovers the potential of moving fungal proteomics beyond the lab and into the clinic to not only offer new avenues for detection and monitoring, but also aiding medical decisions tailored towards treating the individual in a personalised and effective manner.

## Data Availability

Not applicable.
